# Comment on Raine (2019) ‘The neuromoral theory of antisocial, violent, and psychopathic behavior’

**DOI:** 10.12688/f1000research.23346.2

**Published:** 2020-07-21

**Authors:** Hyemin Han

**Affiliations:** 1 Educational Psychology Program, University of Alabama, Tuscaloosa, AL, USA

**Keywords:** psychopathy, antisociality, morality, moral psychology, neuroscience

## Abstract

Raine (2019) reviewed previous research on the neural correlates of antisocial, violent, and psychopathic behavior based on previous studies of neuroscience of morality. The author identified neural circuitries associated with the aforementioned types of antisocial behaviors. However, in the review, Raine acknowledged a limitation in his arguments, the lack of evidence supporting the presence of the neural circuitries. In this correspondence, I intend to show that some of his concerns, particularly those about the insula and cingulate cortex, can be addressed with additional evidence from recent neuroimaging research. In addition, I will propose that the additional evidence can also provide some insights about how to design future neuroimaging studies to examine the functionality of the striatum in the circuitries.

A review article by Raine was published in *Psychiatry Research* in 2019 concerning neuromoral theory of antisocial, violent, and psychopathic behavior ^1^. The author proposed a comprehensive model of the neural network of morality and antisociality to explain the neural-level mechanisms of antisocial behavior. The author referred to previous neuroimaging studies and meta-analyses to identity the aforementioned neural networks and proposed that the prefrontal cortex, amygdala, insula, and anterior cingulate cortex are included in both networks, while the striatum is included in the antisociality network. The author stated two limitations regarding the network model that he proposed: first, the involvement of the insula and cingulate cortex regions in the neural networks could not be sufficiently supported by previous neuroimaging studies and meta-analyses; second, evidence that supports the involvement of the striatum in the morality network is insufficient.

Although Raine raised the aforementioned two concerns regarding the lack of supporting evidence, I suggest that recent research in the field of social neuroscience can provide some supports to his points. Herein, I introduce two supporting findings, one from online-based large-scale analyses of neuroimaging data, and the other from recent neuroimaging experiments focusing on brain circuitries associated with morality. These recent research findings will be able to provide evidence of the involvement of the insula and cingulate cortex, which are involved in both moral and antisocial brain circuitries. Furthermore, some additional evidence might be able to support the involvement of parts of striatal regions in the circuit of antisociality although the evidence is not sufficient to completely address the issue. At least, the additional evidence might be able to provide ideas about potential research questions and hypotheses focusing on the striatum in morality and antisociality.

First, results from large-scale analysis of previous neuroimaging studies provides additional evidence supporting Raine’s model. Thanks to the development of information technology, performing meta-analysis of large-scale neuroimaging data has become feasible. A web-based analysis tool, NeuroSynth, is one example ^2^. NeuroSynth automatically gathers coordinate information that is reported in published neuroimaging articles and performs meta-analysis of the gathered information. A result from a meta-analysis of 87 studies and 2,806 activation foci that are associated with a keyword “moral” demonstrates that the left insula and anterior and posterior cingulate cortices show significant common activity across moral task conditions (see http://neurosynth.org/analyses/terms/moral/ for further details). Moreover, a recently developed analysis tool, NeuroQuery ^3^, also reported the consistent result. Unlike NeuroSynth, which only analyzes published papers’ abstracts, NeuroQuery analyzes the full article texts, so it shows better selectivity compared with NeuroSynth ^3^. It employs machine learning to examine the pattern of neural activation associated with a keyword of interest ^3^. When “morality” was explored by NeuroQuery, both the left and right insula and cingulate cortex showed strong association with the keyword across 68 studies (see https://neuroquery.org/query?text=morality for further details). These results provide evidence that supports the involvement of the insula and cingulate cortices in the neural network of morality based on large-scale data. In fact, the three meta-analysis articles that Raine reviewed meta-analyzed relatively fewer numbers of neuroimaging studies (references ^4– 6^), so he could only partially and tentatively propose the involvement of the insula and cingulate cortex regions in the neuromoral network. Hence, I suggest that Raine’s argument about the involvement of insula and cingulate cortex in the circuitries can be at least partially supported by large-scale neuroimaging data and the result from NeuroSynth and NeuroQuery. Moreover, the reported involvement of the left insula may suggest the possibility of laterality effects that Raine mentioned in his review, although more research that directly focuses on the laterality effects should be conducted.

However, there are several caveats while interpreting these findings. First, although the involvement of the insula was consistently supported by two large-scale neuroimaging data analyses, some of the introduced meta-analyses did not report such a result. One point related to the large-scale analyses that should be considered is that compared with meta-analyses that were conducted with human-involved literature review, such automated analyses, particularly NeuroSynth, simple analyze texts per se so their selectivity might not be optimal. This point warrants further studies that more carefully explore large-scale database. Second, none of the meta-analyses and NeuroSynth directly demonstrated the association between the striatum with morality tasks. This might be due to that the analyzed previous studies were mainly about morality, not antisociality, so they might not be able to well address the neural correlates of antisociality.

Second, recent neuroimaging studies by my research group ^7– 9^ suggest that the insula and striatum regions showed significant activation and interaction with prefrontal and cingulate regions, which were indicated as core regions in the neural network of morality by Raine, in moral task conditions. The author tentatively proposed that increasing evidence may suggest that the striatum can be included in the morality network as well as the antisociality network. The new neuroimaging studies may provide additional evidence that supports the involvement of the striatum in the morality network. Our neuroimaging study from 2014 ^7^ reported that the insula, cingulate cortex, and striatum (e.g. caudate and putamen) were significantly activated when participants were solving moral dilemmas (see Table S1 in 7 for further details). Such findings were also supported by a recent reanalysis with Bayesian inference ^8^. Furthermore, our study from 2016 ^9^ conducted psychophysiological interaction analysis and connectivity analysis based on Granger causality to examine interactions and connections among brain regions in moral task conditions. This study reported that the insula and striatum regions significantly interacted and were connected with other morality-related regions including the medial prefrontal and cingulate cortices.

In addition to these neuroimaging experiments, the result from NeuroQuery might also provide additional supports. Although the size of the identified region was small, NeuroQuery reported that when “morality” was entered, a part of the right putamen, which constitutes the dorsal striatum, showed a significant association with the keyword. Several previous fMRI studies have shown that the putamen was associated with intuition-related moral judgment ^7^, evaluation of moral intentionality of an actor in the relation with potential benefits and losses ^10^, and modulation of moral behavior ^11^. Hence, future studies may focus on the putamen as a possible candidate region to be included in the morality-antisociality circuitries based on these previous findings. Furthermore, although additional investigations with more focused experimental designs are warranted, such findings might be able to provide ideas about the involvement of striatal regions, particularly the putamen, in the morality-antisociality network.

Given the aforementioned additional large-scale analyses and neuroimaging studies, we might have additional evidence that can support the point that the insula and cingulate cortex can be considered as parts of the neural network of morality. Particularly, both large-scale analyses, NeuroSynth and NeuroQuery, supported such a point although the meta-analyses introduced in Raine’s article demonstrated mixed results. One remaining issue is that although the association between the moral-antisocial circuitry and the striatum was partially supported by the additional neuroimaging experiments and NeuroQuery, NeuroSynth and the majority of the meta-analyses did not report such a result. Even if that is the case, the aforementioned results related to the functioning of the striatum might be able to provide researchers with some ideas for research question and hypothesis building. Given that the involvement of the striatum, particularly the putamen, was partially supported, future neuroimaging studies that intend to explore the antisocial circuitry may focus the point with more specialized research designs.

## Data availability

No data is associated with this article.
