# Supplementary material for: Comment on Raine (2019) ‘The neuromoral theory of antisocial, violent, and psychopathic behavior’
Source: F1000Res. 2020 Jul 21;9:274. Originally published 2020 Apr 20. [Version 2] doi: 10.12688/f1000research.23346.2 (PMC7405258; doi:10.12688/f1000research.23346.2)
Supplement: Peer review history

# Peer review: Reviewer response for version 2

**DOI:** 10.5256/f1000research.28132.r67614

**Published:** 2020-08-04

**Authors:** Fernando Barbosa

**Affiliations:** 1 Laboratory of Neuropsychophysiology, Faculty of Psychology and Education Sciences, University of Porto, Porto, Portugal

The author has successfully solved the main limitations of the manuscript in this revised version and I think it can now be endorsed for indexing (although it could benefit from a more thorough proofreading).

Are arguments sufficiently supported by evidence from the published literature or by new data and results?

Partly

Is the conclusion balanced and justified on the basis of the presented arguments?

Partly

Is the rationale for commenting on the previous publication clearly described?

Yes

Are any opinions stated well-argued, clear and cogent?

Yes

Reviewer Expertise:

NA

I confirm that I have read this submission and believe that I have an appropriate level of expertise to confirm that it is of an acceptable scientific standard.

# Peer review: Reviewer response for version 1

**DOI:** 10.5256/f1000research.25769.r65587

**Published:** 2020-07-15

**Authors:** Fernando Barbosa

**Affiliations:** 1 Laboratory of Neuropsychophysiology, Faculty of Psychology and Education Sciences, University of Porto, Porto, Portugal

The author sought to provide additional support concerning the neuronal bases of the neuromoral model of antisocial behavior, initially proposed by Raine and Yang (2006) and recently updated by Raine (2019), who reviewed findings on brain mechanisms underlying moral decision-making and antisocial behavior that were published after the initial proposal. Specifically, this comment was intended to provide evidence of the involvement of the insula, cingulate cortex, and striatum in the circuits of the neuromoral model. To that end, the author uses: (a) NeuroSynth (which is not recent) to obtain meta-analytic data from neuroimaging studies of (supposedly) moral decision-making; and (b) results from his own neuroimaging studies focusing on brain circuitries associated with morality.

 Although the NeuroSynth-based meta-analysis involved 87 studies, it should be noted that: (a) the NeuroSynth performs a semantic search limited to the abstracts of the papers; (b) many of the said studies do not report the involvement of the insula or the cingulate cortex in moral tasks; (c) the NeuroSynth lacks specificity, as moral decision-making is often studied with other constructs, which may have induced the observed brain activity; and (d) automated meta-analyses prevent the careful examination of relevant publication indices, such as the publication/confirmation bias. For these reasons and taking the NeuroSynth results into account, which do not seem substantial *per se*, it is highly questionable whether the author provides stronger evidence than the one compiled by Raine in his own review to support his cautious arguments. It is worth mentioning that Raine refers to four meta-analyses, and most support the role of the cingulate in the neuromoral circuit, but he also explains that a recent meta-analysis by Eres *et al.* (2018) does not. 

 Similarly, Raine reports meta-analytic studies that implicate the insula in morality, but he also explains that other meta-analyses do not (one of them was conducted by the author of this comment), leading to the conclusion that evidence is not sufficient to consider the insula as playing a role in moral decision-making. Moreover, given the fact that recent meta-analyses of morality have not directly implicated the striatum (again, one was authored by Han, 2017), Raine judiciously considers its inclusion in the neuromoral model debatable.

 The studies conducted and reported by the author of this comment do lend additional support to the possible role of the insula and striatum in moral decision-making, but such results do not provide stronger evidence than the meta-analytic findings compiled by Raine. 

 Summing-up, the arguments and results presented here do not make the findings regarding the implication of the cingulate, insula and striatum in moral decision-making any more consistent, nor any less debatable. Both the claim that “the insula, cingulate cortex, and striatum can be considered as parts of the neural network of morality”, and the conclusion that such claim “can be well supported by the analyses that I [the author] introduce here” sound like overstatements. Raine’s cautiousness is still well justified. 

 Minor issues: “circutries”, circutiries, and “evience” are typos.

Are arguments sufficiently supported by evidence from the published literature or by new data and results?

Partly

Is the conclusion balanced and justified on the basis of the presented arguments?

Partly

Is the rationale for commenting on the previous publication clearly described?

Yes

Are any opinions stated well-argued, clear and cogent?

Yes

Reviewer Expertise:

Psychopathy, Antisocial Behavior, EEG/ERP, fMRI, Neuropsychology

I confirm that I have read this submission and believe that I have an appropriate level of expertise to confirm that it is of an acceptable scientific standard, however I have significant reservations, as outlined above.

# Response

**Published:** 2020-07-16

**Authors:** Hyemin Han

**Affiliations:** University of Alabama, USA

**1. **Although the NeuroSynth-based meta-analysis involved 87 studies, it should be noted that: (a) the NeuroSynth performs a semantic search limited to the abstracts of the papers; (b) many of the said studies do not report the involvement of the insula or the cingulate cortex in moral tasks; (c) the NeuroSynth lacks specificity, as moral decision-making is often studied with other constructs, which may have induced the observed brain activity; and (d) automated meta-analyses prevent the careful examination of relevant publication indices, such as the publication/confirmation bias. For these reasons and taking the NeuroSynth results into account, which do not seem substantial per se, it is highly questionable whether the author provides stronger evidence than the one compiled by Raine in his own review to support his cautious arguments. It is worth mentioning that Raine refers to four meta-analyses, and most support the role of the cingulate in the neuromoral circuit, but he also explains that a recent meta-analysis by Eres et al. (2018) does not.

**Response: **Thank you very much for your comments regarding the limitations of NeuroSynth and the point related to the involvement of the cingulate cortex presented in the meta-analysis. In the revised manuscript, I added some descriptions about the limitations of NeuroSynth that warrant a cautious interpretation of results. Simultaneously, as a way to partially address the issue, I introduced NeuroQuery that analyzes the full article texts with Machine learning:

*Moreover, a recently developed analysis tool, NeuroQuery 9, also reported the consistent result. Unlike NeuroSynth, which only analyzes published papers’ abstracts, NeuroQuery analyzes the full article texts, so it shows better selectivity compared with NeuroSynth 9. It employs machine learning to examine the pattern of neural activation associated with a keyword of interest 9. When “morality” was explored by NeuroQuery, both the left and right insula and cingulate cortex showed strong association with the keyword across 68 studies (see https://neuroquery.org/query?text=morality for further details).*

Furthermore, I added a paragraph that discusses some additional caveats while interpreting the results from the large-scale analyses and meta-analyses:

*However, there are several caveats while interpreting these findings. First, although the involvement of the insula was consistently supported by two large-scale neuroimaging data analyses, some of the introduced meta-analyses did not report such a result. One point related to the large-scale analyses that should be considered is that compared with meta-analyses that were conducted with human-involved literature review, such automated analyses, particularly NeuroSynth, simple analyze texts per se so their selectivity might not be optimal. This point warrants further studies that more carefully explore large-scale database. Second, none of the meta-analyses and NeuroSynth directly demonstrated the association between the striatum with morality tasks. This might be due to that the analyzed previous studies were mainly about morality, not antisociality, so they might not be able to well address the neural correlates of antisociality.*

**2. **Similarly, Raine reports meta-analytic studies that implicate the insula in morality, but he also explains that other meta-analyses do not (one of them was conducted by the author of this comment), leading to the conclusion that evidence is not sufficient to consider the insula as playing a role in moral decision-making. Moreover, given the fact that recent meta-analyses of morality have not directly implicated the striatum (again, one was authored by Han, 2017), Raine judiciously considers its inclusion in the neuromoral model debatable.

The studies conducted and reported by the author of this comment do lend additional support to the possible role of the insula and striatum in moral decision-making, but such results do not provide stronger evidence than the meta-analytic findings compiled by Raine.

**Response: **I sincerely appreciate your comments regarding the involvement of the insula and striatum in the circuitries. I added some points from NeuroQuery while toning down my interpretations in general.

*In addition to these neuroimaging experiments, the result from NeuroQuery might also provide additional supports. Although the size of the identified region was small, NeuroQuery reported that when “morality” was entered, a part of the right putamen, which constitutes the dorsal striatum, showed a significant association with the keyword. Several previous fMRI studies have shown that the putamen was associated with intuition-related moral judgment 6, evaluation of moral intentionality of an actor in the relation with potential benefits and losses 10, and modulation of moral behavior 11. Hence, future studies may focus on the putamen as a possible candidate region to be included in the morality-antisociality circuitries based on these previous findings. Furthermore, although additional investigations with more focused experimental designs are warranted, such findings might be able to provide ideas about the involvement of striatal regions, particularly the putamen, in the morality-antisociality network.*

**3. **Summing-up, the arguments and results presented here do not make the findings regarding the implication of the cingulate, insula and striatum in moral decision-making any more consistent, nor any less debatable. Both the claim that “the insula, cingulate cortex, and striatum can be considered as parts of the neural network of morality”, and the conclusion that such claim “can be well supported by the analyses that I [the author] introduce here” sound like overstatements. Raine’s cautiousness is still well justified.

**Response: **Thank you very much for your comments regarding the overall tone of my commentary. I agree with you that the limitations in the existing literature cannot completely confirm the involvement of the aforementioned regions in the circuitries. Thus, I toned down the introduction and conclusion accordingly. Instead of strongly argue that every single issue can be addressed by additional evidence, I proposed that the evidence is somehow suggestive. In other words, I proposed several directions for future research based on the added evidence. Also, as a way to strength my point, I additionally introduced NeuroQuery as well.

*These recent research findings will be able to provide evidence of the involvement of the insula and cingulate cortex, which are involved in both moral and antisocial brain circuitries. Furthermore, some additional evidence might be able to support the involvement of parts of striatal regions in the circuit of antisociality although the evidence is not sufficient to completely address the issue. At least, the additional evidence might be able to provide ideas about potential research questions and hypotheses focusing on the striatum in morality and antisociality.*

*Given the aforementioned additional large-scale analyses and neuroimaging studies, we might have additional evidence that can support the point that the insula and cingulate cortex can be considered as parts of the neural network of morality. Particularly, both large-scale analyses, NeuroSynth and NeuroQuery, supported such a point although the meta-analyses introduced in Raine’s article demonstrated mixed results. One remaining issue is that although the association between the moral-antisocial circuitry and the striatum was partially supported by the additional neuroimaging experiments and NeuroQuery, NeuroSynth and the majority of the meta-analyses did not report such a result. Even if that is the case, the aforementioned results related to the functioning of the striatum might be able to provide researchers with some ideas for research question and hypothesis building. Given that the involvement of the striatum, particularly the putamen, was partially supported, future neuroimaging studies that intend to explore the antisocial circuitry may focus the point with more specialized research designs.*

**4. **Minor issues: “circutries”, circutiries, and “evience” are typos.

**Response:** I appreciate your comments about the typos. In the revised manuscript, I corrected those points.

# Peer review: Reviewer response for version 1

**DOI:** 10.5256/f1000research.25769.r64888

**Published:** 2020-07-07

**Authors:** Pascal Molenberghs

**Affiliations:** 1 Institute for Social Neuroscience (ISN Psychology), Melbourne, Vic, Australia

The author provides a comment on a recently published article by Raine (2019) which is about a neuromodal theory of antisocial, violent, and psychopathic behavior.

Major comments:

 The author makes two main claims:

1. The first claim is about the insula and cingulate cortex involvement in morality. According to the author, there is evidence that the insula and cingulate cortex are involved in morality based on a meta-analysis in Neurosynth with the word “moral”. It is stated that:
  “A result from a meta-analysis of 87 studies and 2,806 activation foci that are associated with a keyword “moral” demonstrates that the left insula and anterior and posterior cingulate cortices show significant common activity across moral task conditions (see http://neurosynth.org/analyses/terms/moral/ for further details).”
  I had a look at the result provided by the link, but I couldn’t see much evidence of insula of cingulate cortex involvement. Maybe there is a voxel or two that overlaps with the left insula and cingulate cortex but there is barely any evidence of this. Also, although I like the program Neurosynth, its data is quite messy because it just automatically scans studies based on a word (in this case “moral”). The algorithm will often make mistakes that can be overcome by a carefully conducted “manual” (i.e., not automatic) neuroimaging meta-analysis. Therefore, I wouldn’t trust a Neurosynth meta-analysis more than a thoroughly conducted manual neuroimaging meta-analyses on the topic. Given that several manual neuroimaging meta-analyses on the topic of morality couldn’t find any evidence of insula and cingulate cortex involvement, I don’t think that this Neurosynth meta-analysis (that barely shows any evidence of insula or cingulate cortex activity) is now the crucial missing evidence that we have been waiting for.
2. The second claim is that the striatum is involved in the morality network. Here the author uses three neuroimaging studies by his own research group. The three studies seem to be based on the same data of around 16 (sic) participants. I don’t doubt that the author is familiar with their own study and that striatum was activated but again, I don’t think this is the convincing evidence to finally claim that the striatum is consistently involved in morality. If the author wants to make the point about the striatum (or the insula and cingulate cortex), they should do a thorough look through the neuroimaging morality literature to argue their case.

 

 In short, I don’t think the comment adds much to the literature.

Minor comments:

 1) circutiries => circuitries

 2) recently invented => Neurosynth has been around since 2011

Are arguments sufficiently supported by evidence from the published literature or by new data and results?

No

Is the conclusion balanced and justified on the basis of the presented arguments?

No

Is the rationale for commenting on the previous publication clearly described?

Yes

Are any opinions stated well-argued, clear and cogent?

Yes

Reviewer Expertise:

fMRI, morality, meta-analyses

I confirm that I have read this submission and believe that I have an appropriate level of expertise to state that I do not consider it to be of an acceptable scientific standard, for reasons outlined above.

# Peer review: Reviewer response for version 1

**DOI:** 10.5256/f1000research.25769.r62632

**Published:** 2020-05-12

**Authors:** Ji-Won Hur

**Affiliations:** 1 Department of Psychology, Korea University, Seoul, South Korea

The current literature is a well written concise commentary. The title and the abstract precisely summarize the content of the article. The authors provided evidence supporting Raine’s model on the presence of the neural circuitry of antisociality. The concluding paragraph also clearly reflects the author's argument. This commentary would be useful to design or establish further research or hypothetical model regarding the insula, cingulate cortex, and striatum regions as parts of the neural network of morality or antisociality.

 (there are some typos (circutries) in the manuscript).

Are arguments sufficiently supported by evidence from the published literature or by new data and results?

Yes

Is the conclusion balanced and justified on the basis of the presented arguments?

Yes

Is the rationale for commenting on the previous publication clearly described?

Yes

Are any opinions stated well-argued, clear and cogent?

Yes

Reviewer Expertise:

Psychopathology, Social neuroscience.

I confirm that I have read this submission and believe that I have an appropriate level of expertise to confirm that it is of an acceptable scientific standard.

# Response

**Published:** 2020-07-16

**Authors:** Hyemin Han

**Affiliations:** University of Alabama, USA

Thank you very much for your kind comments. First of all, I corrected the typos in the manuscript that you mentioned. Second, following your suggestion and other reviewers' points, I added some points related to how my commentary can provide insights about how to develop further research questions and hypotheses.
